# Positive rate of wheat allergens in the Chinese allergic population: a systematic review and meta-analysis

**DOI:** 10.1038/s41598-023-37648-2

**Published:** 2023-06-29

**Authors:** Fengmei Yang, Xinyi Zhao, Wenfeng Liu, Bo Zhou, Lili Deng, Hongbing Chen, Zhuo Zhang, Lin Zhou

**Affiliations:** 1 School of Public Health, Shenyang Medical College, Shenyang, 110034 China; 2 State Key Laboratory of Food Science and Technology, Nanchang University, Nanchang, 330047 China; 3 College of Integrated Chinese and Western Medical, Liaoning University of Traditional Chinese Medicine, Shenyang, 110033 China

**Keywords:** Health care, Public health, Epidemiology

## Abstract

In recent years, the prevalence of allergic diseases has increased significantly, causing great concern, and wheat, as one of the top 8 food allergens, is a common allergy trigger. Nevertheless, reliable estimates of the positivity rate of wheat allergens in the allergic population in China are still lacking. The systematic review and meta-analysis aims to evaluate the positive detection rate of wheat allergens in the Chinese allergic population and further provide a reference for the prevention of allergy. CNKI, CQVIP, WAN-FANG DATA, Sino Med, PubMed, Web of Science, Cochrane Library, and Embase databases were retrieved. Related research and case reports about the positive rate of wheat allergen in the Chinese allergic population published from inception to June 30, 2022, were searched, and meta-analysis was performed using Stata software. The pooled positive rate of wheat allergens and 95% confidence interval were calculated by random effect models, and the publication bias was evaluated using Egger’s test. A total of 13 articles were included for the final meta-analysis, in which wheat allergen detection methods involved only serum sIgE testing and SPT assessment. The results showed that the wheat allergen positivity detection rate in Chinese allergic patients was 7.30% (95% CI 5.68–8.92%). Subgroup analysis showed that the positivity rate of wheat allergens was influenced by region, but hardly by age and assessment method. The positive rates of wheat allergy in the population with allergic diseases were 2.74% (95% CI 0.90–4.58%) and 11.47% (95% CI 7.08–15.87%) in southern and northern China, respectively. In particular, the positive rates of wheat allergens were greater than 10% in Shaanxi, Henan and Nei Mongol, all of which belong to the northern region. These results suggest that wheat allergens are an important cause of sensitization in allergic populations from northern China, and therefore attention should be paid to early prevention in high-risk populations.

## Introduction

Wheat is one of the three most recognized grains in the world and is widely grown and eaten by people all over the world. Moreover, wheat is highly nutritious and palatable and can be processed into a variety of staple foods such as bread, noodles, pizza, snack, and beverages such as beer^1,2^. However, wheat is increasingly acknowledged as a trigger for immune-mediated food allergies, including immuno-globulin E (IgE) and non-IgE-mediated food allergies^1,3^. Wheat allergy is typically characterized by the involvement of T helper type 2 (Th2) cells, which secrete Th2 cytokines such as IL-4, IL-13 and IL-5 in the allergic response^4^. Concerning IgE-mediated wheat allergy, Th2 inflammation can produce IgE antibodies by B cells specific to a certain ingredient. Non-IgE-mediated wheat allergy is characterized by chronic eosinophilic inflammation induced by Th2 lymphocytic response, mainly including eosinophilic esophagitis (EoE) or eosinophilic gastritis (EG)^4^.

The major allergens for wheat allergy are mainly composed of gliadin, α-amylase/trypsin inhibitor, peroxidase, serine proteinase inhibitor, α-purothionin, and so on^5^. IgE-mediated wheat allergy is immediate and even can be severe and life-threatening, which presents with a variety of symptoms including wheat-dependent exercise-induced anaphylaxis (WDEIA), baker’s asthma, allergic rhinitis, contact urticaria, atopic dermatitis (AD), asthma, and leprosy skin diseases^6,7^. The most common symptoms of IgE-mediated wheat allergy, which usually appear within minutes to hours after exposure, involve gastrointestinal symptoms (nausea, abdominal pain, vomiting, diarrhea), skin (pruritus, eczema, redness), respiratory (rhinitis, asthma), circulatory (flushing, angioedema), and cerebral (thought disturbance, headache, dizziness)^1,6,8^. For non-IgE-mediated wheat allergy, the typical manifestations are dyspepsia, diarrhea, vomiting, arthralgia and headache, which may appear hours or days after ingestion of the allergen^9^. However, the manifestation of clinical symptoms of allergic reactions is related to a variety of factors: the patient’s age, region, immune status, amount of allergen, allergenic protein, route of sensitization, and environmental factors^10^.

In western countries, the prevalence of wheat-based food allergy from the epidemiological survey population is likely in the range of 0.2% to 1%, which varies according to age and region^1,2^. Children have a higher incidence of wheat allergy than adults and are more likely to develop an allergy if wheat is introduced after 6 months of life, however, approximately 65% of Children can outgrow allergies by age 12^4^. In Europe, the prevalence of wheat allergy is less than < 1%. A systematic epidemiological evaluation by Zuidmeer et al.^11^ displayed that the prevalence of wheat allergy confirmed by oral food challenge (OFC) as a diagnostic criterion varied between 0.2 and 0.5% in allergic patients under 14 years of age^12^. In the UK, the children’s prevalence of wheat allergy was 0.48% based on positive OFC^11^. In US children, a prevalence of wheat allergy above 3% was reported according to the skin prick test (SPT) in epidemiological surveys, even though it is more likely to be estimated at 0.2% to 1% in allergic patients^1,13^. In a general population aged 0–6 years from Japan, the proportion of positive serum IgE antibodies to wheat SPT/ω-5 gliadin was 0.37%^14^. In addition, it is noteworthy that many studies have confirmed that the prevalence of self-reported wheat allergy is higher than that of wheat allergy determined by the OFC. For example, A systematic study conducted by Nwaru et al.^15^ suggested that a self-reported prevalence of wheat allergy was 1.5%.

In the last 20 years, there has been a gradual increase in reported cases of wheat allergy in China. Wheat (15.2%) was found to rank third among the most common allergens in 992 children seen in the dermatology department of Shengjing Hospital of China Medical University from October 2016 to March 2019^16^. Another study that statistically analyzed the combined data of 19,453 inhalation allergens and 20,556 food allergens patients attending Tianjin Children’s Hospital from January 2019 to March 2020 showed that wheat (18.92%) also ranked third among the most common allergens^17^. A retrospective study of 1,952 anaphylactic shocks in 907 patients showed that food was the most common cause (77%), among which wheat was responsible for 20% of food-induced anaphylaxis in adolescents (10–17 years) and 42% of anaphylaxis in adults (18–50 years)^18^.

However, there is still a lack of integrated analysis of cases and studies related to the positive rate of wheat allergens in the Chinese allergic population. In this context, a single-group rate meta-analysis was used to integrate the reported cases and studies related to wheat allergens, following analyzing the positive rate of the Chinese allergic population, to provide a scientific basis for the prevention of this disease.

## Materials and methods

The study was unregistered and conducted by the Preferred Reporting Items for Systematic Review and Meta-analysis (PRISMA) guidelines and the PRISMA 2020 checklist^19^.

### Study selection and data extraction

Peer‑reviewed cases and studies about the positive rate of wheat allergens in the Chinese allergic population written in Chinese and English from inception to June 30, 2022, were retrieved from the following electronic databases: China National Knowledge Infrastructure (CNKI), Chongqing VIP (CQVIP), WAN-FANG DATA, Sino Med, PubMed, Web of Science, Cochrane Library, Embase databases. A search strategy combining medical subject headings (MeSH) and natural language text words was used (Supplementary Information 1). The following search keywords or phrases were employed to find relevant articles: (“allergic diseases” OR “anaphylactic reaction” OR “reaction, anaphylactic”) AND (“wheat allergens” OR “allergen, wheat” OR “wheat allergen”) AND (“positive rate” OR “positive detection rate” OR “detectable rate” OR “detection rate”) AND (“China” OR “Chinese”). Finally, all studies classified as positive for wheat allergens in the Chinese allergic population were screened. The data extraction included, but was not limited to, the following information: the first author’s name, year of publication, study design, age range, sample size, study population, gender, assessment method, research location, diagnostic criteria, and main results.

### Inclusion criteria and exclusion criteria

To meet the analysis requirements and reduce deviation, eligible studies fulfilled the following criteria: (i) all study subjects were Chinese population and with clinically confirmed allergic disease or suspected allergy; (ii) literature relates to geographical location in mainland China, Hong Kong, Macau and Taiwan; (iii) diagnostic methods were based on a questionnaire, clinical diagnosis, double-blind placebo-controlled oral food challenge (DBPOFC)^20^, serum specific IgE test (sIgE), skin prick test (SPT), atopy patch test (APT), intradermal test, oral food challenge (OFC)^21–23^; (iv)the total number of participants and the number or the incidence of positive wheat allergen were available for the included study; (v) the study design types were cross-sectional, retrospective, cohort studies and comparative studies.

The exclusion criteria were as follows: (i) non-Chinese population; (ii) population without a clear clinical diagnosis of allergic disease; (iii) repeated publications; (iv) systematic reviews, conference reports, single case reports other non-original data studies, and animal and cellular studies; (v) articles were unable to be retrieved; (vi) the total number of participants and the number or the incidence of positive wheat allergen were unavailable.

During the inclusion and exclusion process, Endnote X9 software was employed to screen the literature and remove duplicates. Subsequently, titles and abstracts of non-duplicate papers were screened, followed by reading the full text to identify included and excluded studies. Literature was screened independently by two researchers based on inclusion and exclusion criteria. If there was any inconsistency in the judgment of the literature, this was resolved by consensus or consultation with a third reviewer.

### Literature quality evaluation and risk of bias assessment

Cross-sectional Research Quality Rating Scale developed by the Agency for Health Care Research and Quality (AHRQ) was used to evaluate the quality of the included literature. The responses were “yes”, “no”, and “unclear”: “yes” was a score of 1, and “no” or “unclear” was a score of 0. A total score of less than or equal to 5, 6–7 and greater than or equal to 8 were considered as low-quality literature, moderate quality literature, and high-quality literature, respectively^24^. All of the literature was independently evaluated by two reviewers, and any discrepancies were resolved by consensus, with arbitration by a third reviewer if necessary.

### Data analysis

Meta-analysis was performed by Stata/SE 12.0 software, and the pooled positive rate of wheat allergen in the Chinese allergic population and the corresponding 95% confidence interval (CI) were estimated. The heterogeneity of studies was evaluated using the Q and *I*^2^ test. If there was significant heterogeneity among the included literature (*P* < 0.05, *I*^2^ ≥ 50%), a random-effects model was adopted to analyze the positivity rate of wheat allergens. Otherwise, a fixed-effects model was employed. When the heterogeneity was statistically significant (*P* < 0.05, *I*^2^ ≥ 50%), further subgroup analyses and sensitivity analyses were conducted to obtain the causes of heterogeneity. In addition, Egger’s test was used to evaluate the publication bias.

## Results

### Study selection

**Figure 1:**
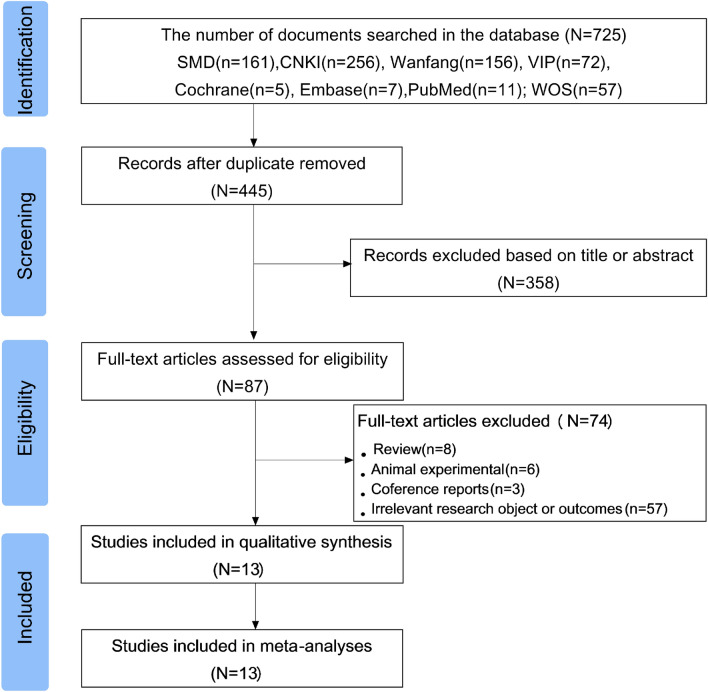
Flow diagram of screening the literature. A total of 13 papers published between 2008 and 2022 were finally included in this study for analysing the positive rate of wheat allergens in the Chinese allergic population.

The PRISMA flowchart shown in Fig. 1 illustrates the process of literature selection and screening. Based on the search strategy, a total of 725 studies on the prevalence of wheat allergen positivity in the Chinese allergic population were eventually retrieved from eight selected electronic databases. After eliminating duplicates, the titles and abstracts of 445 articles were further confirmed and 358 records that did not meet the inclusion criteria were excluded. Subsequently, the eligibility of 87 articles was evaluated through full-text reading. According to the inclusion and exclusion criteria, 13 studies of Chinese allergic populations were finally selected.

### Characteristics of the included studies

As can be seen from Table 1, a total of 13 papers were included in this study, published from 2008 to 2022. The meta-analysis involved a total of 55,762 persons with allergic diseases, of which the number of positive wheat allergens was 2112 cases. Among the studies, nine were conducted in northern China and five in the southern region of China. Both male and female participants were included in the study. Furthermore, the method used for wheat allergen determination involved sIgE and SPT. The included literature covered respiratory allergic diseases, allergic rhinitis, food allergy and other allergic diseases.

The quality of the literature included in the final study was evaluated according to the AHRQ scale, as detailed in Table 2. 13 papers included in this meta-analysis^25–37^ scored 6–7 and were rated as “moderate quality”.

**Table 1:** Characteristic information of the literature included in the meta-analysis.

First author	Year	Study design	Province	Study population	Gender		Age of subjects	Positive/case	Total/case	Positive rate (%)	Assessment method
					Female	Male					
Chen ZY^27^	2017.5–2019.12	Cross-sectional study	Zhejiang	Any food allergy	115	239	0–18 years old	28	354	7.91	sIgE
Du WJ^28^	2018.1–2020.12	Cross-sectional study	Henan	Respiratory allergic diseases	210	194	18–79 years old	73	404	18.07	sIgE
Li J^29^	2019.1–2020.1	Cross-sectional study	Beijing	Any allergic diseases	364	440	0–14 years old	34	804	4.23	sIgE
Liu F^31^	2020.2–2021.2	Cross-sectional study	Shaanxi	Respiratory allergic diseases	235	279	0.5–14 years old	123	514	23.93	sIgE
Qin P^32^	2006.9–2009.8	Cross-sectional study	Liaoning	Any allergic diseases	49	72	1–14 years old	11	121	9.09	SPT
Rina^33^	2017.1–2019.12	Cross-sectional study	Nei Mongol	Allergic rhinitis	613	827	0–18 years old	184	1440	12.78	sIgE
Song X^34^	2010.1–2020.12	Cross-sectional study	Beijing	Suspected allergic disease	16,828	28,912	0–17 years old	1417	45,746	3.10	sIgE
Sun ZY^35^	2006.5–2007.5	Cross-sectional study	Liaoning	Any allergic diseases	669	390	1–84 years old	112	1059	10.58	SPT
Zhao XY^37^	2015.1–2018.1	Cross-sectional study	Guangdong	Allergic dermatitis	1496	1064	0–77 years old	20	2560	0.78	SPT
Li WJ^30^	2016.1–2018.12	Cross-sectional study	Guangdong	Any allergic diseases	901	1104	≥ 18 years old	99	2005	4.94	sIgE
Cao W^26^	2013.3–2015.3	Cross-sectional study	Shaanxi	Systemic contact dermatitis	25	11	33.2 years old	4	36	11.11	sIgE
Bao CC^25^	2012.1–2020.8	Cross-sectional study	Guangdong	Allergic asthma	144	88	18–80 years old	1	232	0.43	sIgE
Xu YS^36^	2010.1–2012.10	Cross-sectional study	Fujian	Any allergic diseases	150	301	0–14 years old	6	451	1.33	sIgE

**Table 2:** Quality evaluation and risk of bias assessment of included literature.

First author	1	2	3	4	5	6	7	8	9	10	11	Score
Chen ZY^27^	Yes	Yes	Yes	Yes	No	Yes	Yes	No	–	Yes	–	7
Du WJ^28^	Yes	Yes	Yes	Yes	No	Yes	No	No	–	Yes	–	6
Li J^29^	Yes	Yes	Yes	Yes	No	Yes	No	No	–	Yes	–	6
Liu F^31^	Yes	Yes	Yes	Yes	No	Yes	No	No	–	Yes	–	6
Qin P^32^	Yes	Yes	Yes	Yes	No	Yes	Yes	No	–	Yes	–	6
Rina^33^	Yes	Yes	Yes	Yes	No	Yes	No	No	–	Yes	–	6
Song X^34^	Yes	Yes	Yes	Yes	No	Yes	No	No	–	Yes	–	6
Sun ZY^35^	Yes	Yes	Yes	Yes	No	Yes	Yes	No	–	Yes	–	7
Zhao XY^37^	Yes	Yes	Yes	Yes	No	Yes	No	No	–	Yes	–	6
Li WJ^30^	Yes	Yes	Yes	Yes	No	Yes	No	No	–	Yes	–	6
Cao W^26^	Yes	Yes	Yes	Yes	No	Yes	Yes	No	–	Yes	–	7
Bao CC^25^	Yes	Yes	Yes	Yes	No	Yes	Yes	No	–	Yes	–	7
Xu YS^36^	Yes	Yes	Yes	Yes	No	Yes	No	No	–	Yes	–	6

Replace unclear; 1 define the source of information(survey, record review); 2 list inclusion and exclusion criteria for exposed and unexposed subjects(cases and controls) or refer to previous publications; 3 indicate time period used for identifying patients; 4 indicate whether or not subjects were consecutive if not population-based; 5 indicate if evaluators of subjective components of study were masked to other aspects of the status of the participants; 6 describe any assessments undertaken for quality assurance purposes (e.g., test/retest of primary outcome measurements); 7 explain any patient exclusion from analysis; 8 describe how confounding was assessed and/or controlled; 9 if applicable, explain how missing data were handle in the analysis; 10 summarize patient response rates and completeness of data collection; 11 clarify what follow-up, if any, was expected and the percentage of patients for which incomplete data or follow-up was obtained.

### Pooled positive rate of wheat allergen

**Figure 2:**
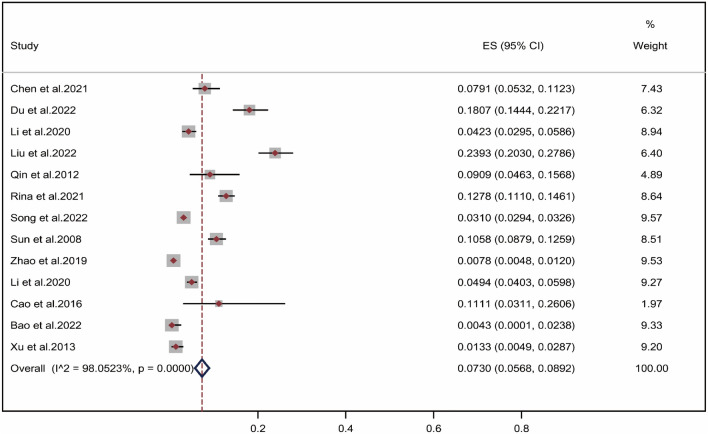
Pooled point positive rate of wheat allergen in the Chinese allergic population. Markers represent percentages and 95%CI, and boxes represent the size of the study.

As shown in Fig. 2, due to heterogeneity in the included literature^25–38^ (*I*^2^ = 98.05% > 50%, *P* = 0.000 < 0.001), a random-effects model was used for meta-analysis. Meta-analysis of single-group rates showed that pooled positive rate of wheat allergen in the Chinese population with allergic disease was 7.30% (95% CI 5.68–8.92%).

### Subgroup Analysis by Geographical Region

**Figure 3:**
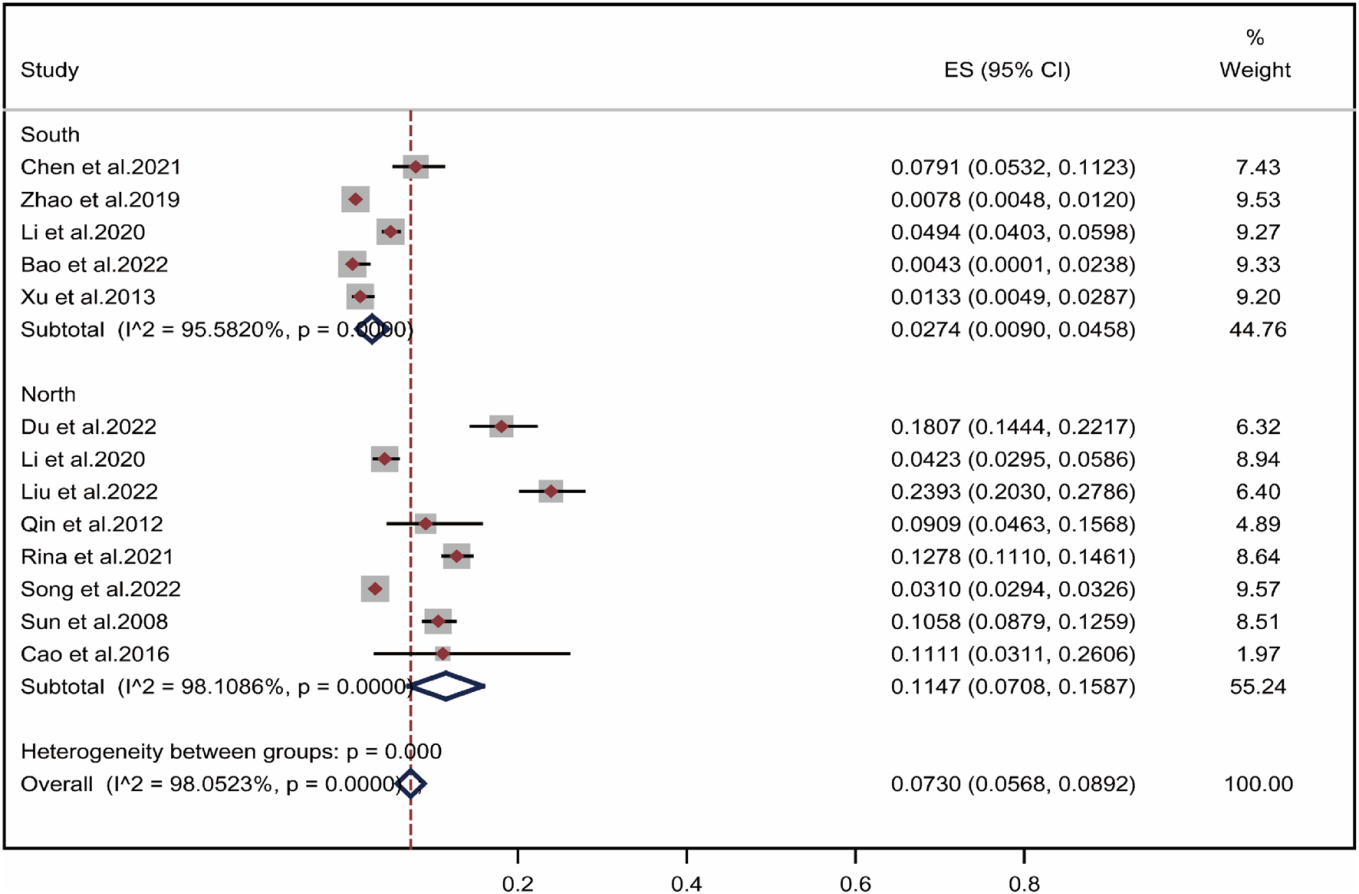
Subgroup Analysis by Geographical Region. Pooled point positive rate of wheat allergen by geographical region in the Chinese allergic population. Markers represent percentages and 95% CI, and boxes represent the size of the study.

**Figure 4:**
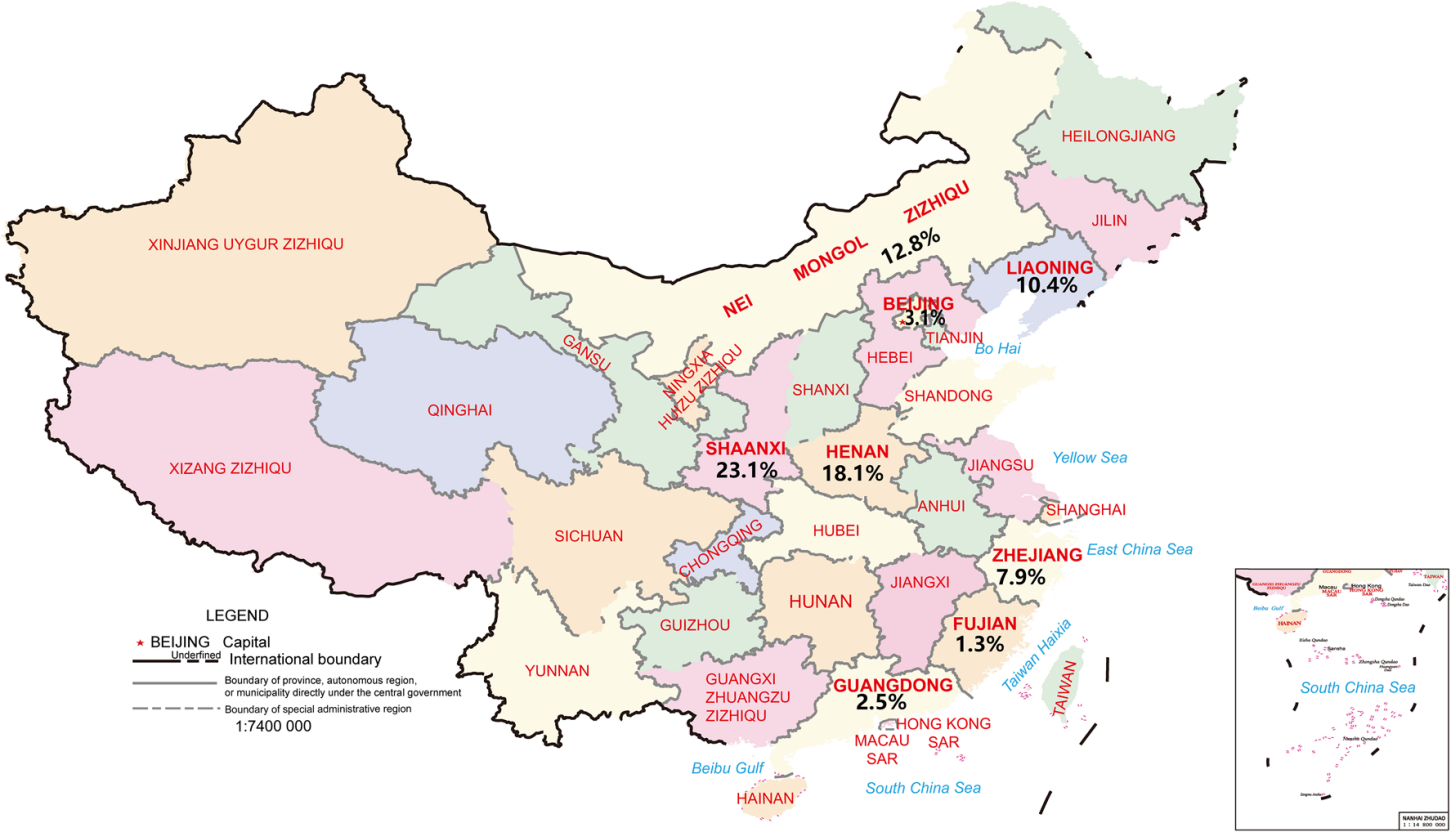
Geographical distribution of positive rate of wheat allergen in the Chinese allergic population.

The literature was analyzed by subgroups of regions according to the origin of the study population, which was divided into the south and the north using the Qinling and Huaihe River as the boundary. As shown in Fig. 3, the positive rate of wheat allergen in allergic population in southern China^26,28,30–35,38^ was 2.74% (95% CI 0.90–4.58%), while the positive rate of wheat allergen in northern China^25,27,29,36,37^ was 11.47% (95% CI 7.08–15.87%). The results revealed that the rate of wheat allergen positivity varied by region, with a significantly higher rate among allergic patients in northern China. The geographical distribution of the positive rate of wheat allergen among the Chinese allergic population was presented in Fig. 4. In Shaanxi, which is in the northwest region of China, the positive rate of wheat allergens in the allergic population was 23.1%^26,31^, while the positive rate of wheat allergens in Beijing was only 3.1%^29,34,38^. In East China, the positive rate of wheat allergens among people with allergic diseases was 7.9% in Zhejiang^27^, but only 1.3% in Fujian^36^. Although limitations in the number of inclusion and assessment methods caused either high or low pooled positive rate of wheat allergens in some areas, these results indicate that the positive rate of wheat allergens in Chinese allergic population was higher in northern China than in southern China.

### Subgroup analysis by assessment methods

**Figure 5:**
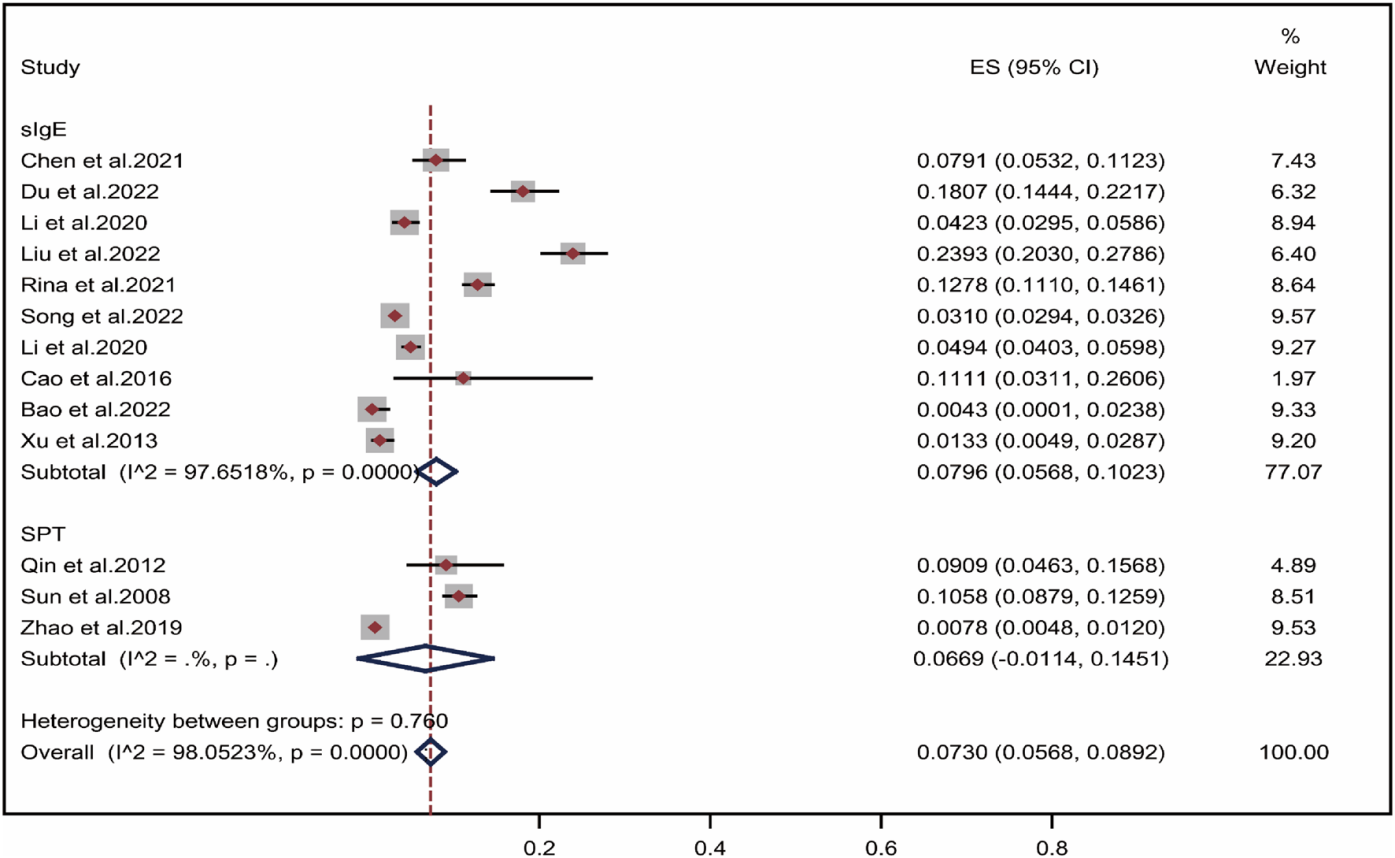
Pooled point positive rate of wheat allergen by assessment methods in the Chinese allergic population. Markers represent percentages and 95% CI, and boxes represent the size of the study.

As shown in Fig. 5, two assessment methods, SPT and sIgE, were included for the evaluation of the positive rate of wheat allergens in the Chinese allergic population. The overall positive rate of wheat allergens using sIgE and SPT was 7.96% (CI 5.68–10.23%)^25–31,33,34,36,38^and 6.69% (CI − 1.14% to 14.51%)^32,35,37^, respectively. Overall, the results of the subgroup analysis of the assessed methods showed no significant difference in the rate of wheat allergen positivity using sIgE and SPT (*P* = 0.760).

### Subgroup analysis by age

**Figure 6:**
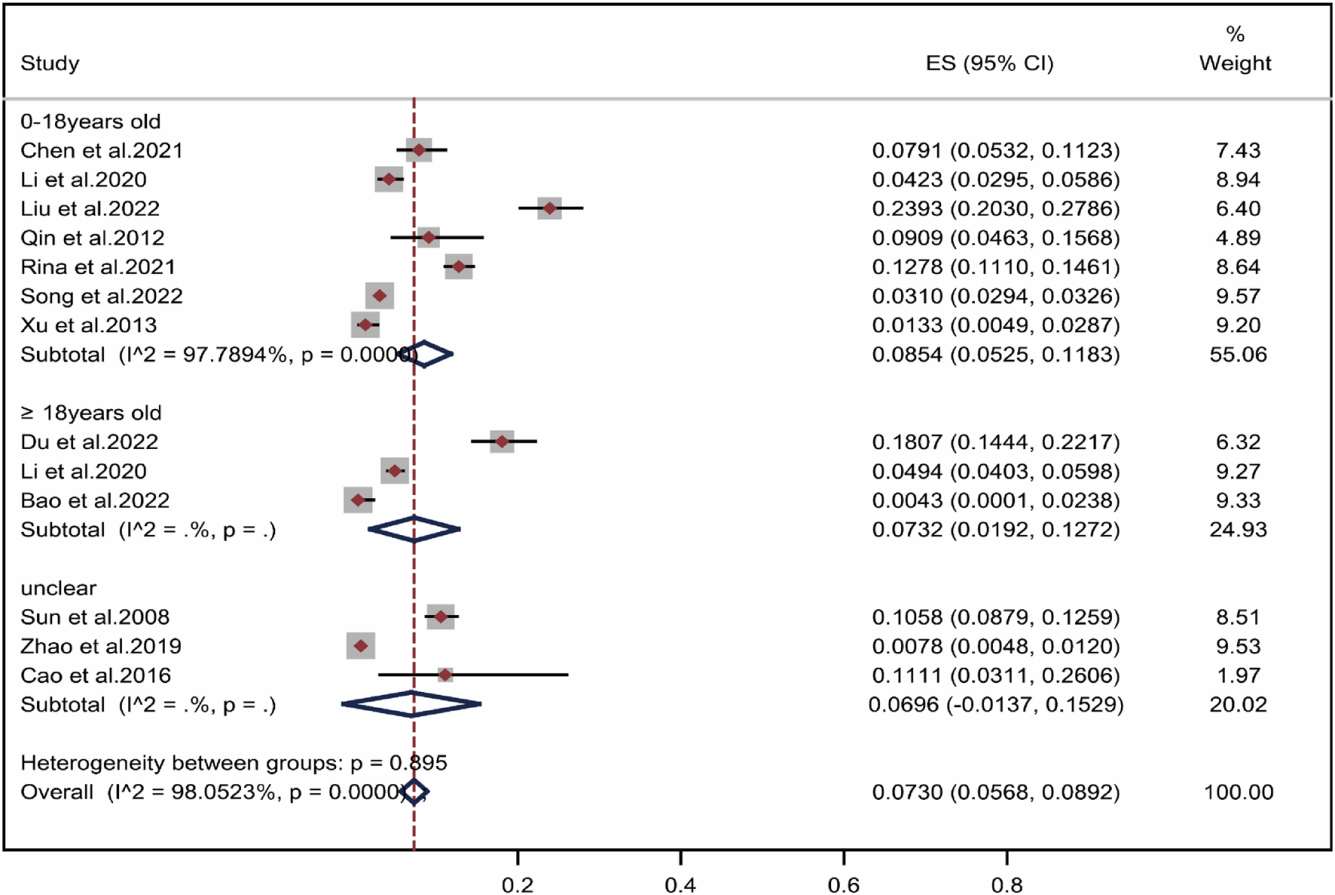
Pooled point positive rate of wheat allergen by age in the Chinese allergic population. Markers represent percentages and 95% CI, and boxes represent the size of the study.

It can be seen from Fig. 6, the positive rate of wheat allergens in the Chinese allergic population showed an increasing trend with age. The wheat allergen positivity rate was increased from 8.54% (CI 5.25–11.83%) in aged 0–18 years^27,29,31–34,36^ to 7.32% (CI 1.92–12.72%) in adults aged 18 years and older^25,28,30,38^. The results in Fig. 6 suggested that there was no significant difference in the rate of wheat allergen positivity between 0 and 18 years and adults aged 18 years and older (*P* = 0.895).

### Sensitivity analysis and publication bias

**Figure 7:**
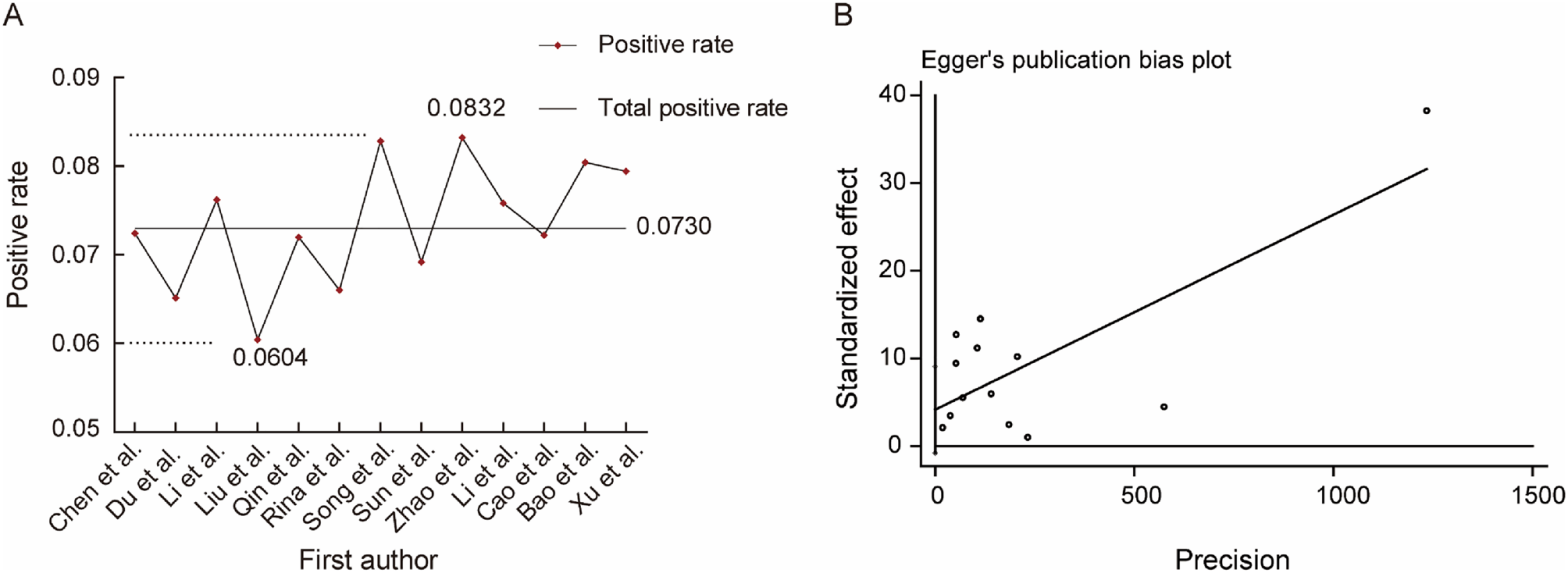
Sensitivity Analysis and Publication Bias. (**A**) sensitivity analysis of the literature about the Chinese allergic population; (**B**) Egger’s Test.

To explore the extent of each study’s effect on the combined effect size and the robustness of the results, sensitivity analyses were therefore performed by excluding the literature one by one for articles included in Chinese allergic population. The results showed that the positive rate of wheat allergens was affected by the exclusion of some literature. This phenomenon may be related to the quality of literature included in the study, different dietary preferences in different regions, and different methods of wheat allergen assessment. It can be seen from Fig. 7A, that the pooled positive rate of wheat allergens in the Chinese population with allergic diseases was 7.30%. However, it presented that the positive rate of wheat allergens was 8.32% after removing the study of Zhao et al.^34^ who tested 2 560 cases of Chinese allergic population, while the positive rate of wheat allergens was 6.04% after removing the study of Liu et al.^31^ who performed sIgE on 514 children. Therefore, the results of this study are somewhat unstable, and subsequent studies should try to include high-quality literature as well as literature with small differences in sample size for a more comprehensive study. The analysis of publication bias was performed using Egger’s test as shown in Fig. 7B and the results suggested that there was no publication bias in the Meta-analysis (*P* = 0.090 > 0.05).

## Discussion

As the incidence of allergic diseases shows an increasing trend, there has been a growing concern about allergies. Dietary ingredients, pollen and mites can all be allergens that trigger allergic diseases^39^. Wheat, one of the top eight food allergens, is present in a variety of staple foods, snacks and beverages, and is also a common allergy trigge^1–3^. For people suffering from allergic diseases, it is essential to pay attention to diet and environment to prevent the occurrence of allergies. The study systematically evaluated and analyzed the positive rate of wheat allergens in the Chinese allergic population with allergic symptoms such as urticaria, asthma, allergic rhinitis, etc. 13 screened articles were finally included and a total of 55,762 patients with allergic disease were investigated, among which 2112 samples were positive for wheat allergens. The results showed that the positive rate of wheat allergen in the Chinese allergic population was 7.30% (95% CI 5.68–8.92%), which will provide some reference for allergy prevention from a food perspective.

Allergy may be related to factors such as dietary habits and geographical location, but also relative humidity, UV light exposure and differences in vitamin D exposure, and so on^40,41^. Further subgroup analyses of the positive rate of wheat allergen in the Chinese populations were performed according to the southern and northern regions of China, using the Qinling and Huaihe River as the boundary. The results suggested that the positive rate of wheat allergen was higher in the northern regions of China than in the south in the allergic population (11.47% vs. 2.47%), which is consistent with the findings of Jiang et al*.*^18^. It is important to mention that the high prevalence in some provinces is due to dietary habits and environmental factors. For example, in a study on wheat allergens testing in Shaanxi Province, a high positive rate of wheat allergens in the allergic population was 23.93% (Table 1)^31^. It is well known that wheat is the main food in the northern regions of China, such as Liaoning, Shandong, Henan, Shaanxi, etc. Moreover, the north is the main wheat-growing area in China. This environmental factor is also responsible for the high incidence of wheat allergy in the north. Telloli et al.^42^ found that the most common component of suspended air-borne particulate matter in wheat growing areas was found to be Aspergillus spores, and exposure and inhalation of these spores may increase the risk of allergic diseases. In addition, a study in Gansu, northern China by He et al.^43^ found that the presence of flour mite parasites in home-stored wheat increased the risk of developing allergic diseases.

In the Chinese allergic population, the positive rate of wheat allergens was 7.30%, which is consistent with a study performed by Chung et al*.*^44^ found the prevalence of wheat allergy was 7.1% in Korean patients with chronic urticaria. Another Asian study of patients with allergic rhinitis found a wheat allergy rate of 10.5% in children (0–4 years), which was higher than our findings, presumably because of the younger age of the population in that study^44^. What’s more, the positive rate of wheat allergens in the allergic population was indeed much higher than in the general population. The rate of wheat allergen positivity in the Chinese allergic population was 7.30%, which was higher than the rate in the general population of Western countries (0.2% to 1%)^1,2^. The prevalence of wheat allergy in Japanese adults was found to be 0.21% using a combination of questionnaires, skin prick tests, and serum ω-5 alcoholic protein-specific IgE assays^45^, which was lower than the positive rate of wheat allergens in the Chinese allergic population. The reasons for these results may be related to dietary habits, vitamin D intake, relative humidity, UV exposure, and other factors^40,41^. In addition, some studies have shown that Asian children had a higher risk of wheat allergy than non-Asian children born in Australia, regardless of whether they were born in Asia or Australia^46^. Therefore, it may be speculated that the prevalence of wheat allergy in China may also be related to genes and parental wheat sensitivity.

According to diagnostic modalities of allergen, subgroup analyses were conducted. Our results showed that the positivity rate of wheat allergens determined by the sIgE was 7.96%, while the positivity rate determined by the SPT was 6.69%. Moreover, significant heterogeneity between studies was observed, which may indicate important differences between studies in terms of study design and methods used to measure the rate of wheat allergens positivity. Sample sizes also varied considerably between studies, and in some studies enough information for accurate calculations was not provided, thus suggesting a potential selection bias in some literature. These methodological limitations would affect the estimates of wheat allergy frequencies reported in the pooled analyses. Therefore, it is recommended that caution should be exercised in interpreting these results.

Different methods used to assess wheat allergy incidence vary widely because the definition of food allergy varies considerably based on each method, for example, the threshold for defining IgE or SPT sensitization to food allergens in the included studies. Moreover, cross-reactivity between different allergens may lead to varying results in assessing the incidence of wheat allergy. Many individuals are sensitive to wheat but can tolerate wheat exposure, especially grass pollen sensitive individuals. Previous studies reported that grass pollen sensitive individuals had allergen-specific IgE for cereal sources due to cross-reactivity between wheat flour and grass pollen as a result of common IgE epitopes in both wheat flour and grass pollen proteins^47–49^.

Methods for the evaluation of IgE-mediated wheat allergy include self-report, SPT, sIgE sensitization, and OFC. Although the self-reported method is easy, convenient, sustainable and inexpensive, the individuals’ perceptions of wheat allergy can influence the diagnosis because they may misunderstand the intolerance or toxicity of the allergy^50^. SPT is rapid, highly sensitive, with high negative predictive values and allows testing with fresh foods, but has low specificity and positive predictive values, and requires stopping antihistamines, clear skin, free from eczema and a trained professional to perform the test^51^. sIgE is quantitative, highly sensitive, standardized, with a high negative predictive value and in vitro, but has lower specificity and is more expensive than SPT^51^. Testing for suspected food allergy is best done with access to an OFC, which is currently the gold standard test for diagnosing food allergy. However, OFC is not readily accepted by patients and their families because it is time-consuming and carries the risk of acute allergic reactions of unpredictable severity^1,52^.

Moreover, basophil activation tests and mast cell activation tests are also used for the clinical diagnosis of food allergy. These tests have high specificity and allow in vitro testing without the risk of allergic reactions, but they are more expensive than sIgE or SPT and are not available in most clinical settings. In addition, clinical diagnosis would be achieved based on the assessment of early warning markers of food allergy, such as cytokine^53^, Treg cell and T cell number and function^54,55^. Further, machine learning-based bioinformatics approaches^56^ and multi-omics technologies^57^are expected to accurately diagnose food allergies in high throughput. In conclusion, the diagnosis of wheat allergy requires use of various combinations of these measures to improve the accuracy and generalizability of the diagnosis.

In our meta-analysis, there was no significant difference between the age groups. Wheat allergen positivity showed an increasing trend from 8.54% (CI 5.25–11.83%) in 0–18 years of age to 7.32% (CI 1.92–12.72%) in adults over 18 years of age. Our findings confirm a previous study that the prevalence of food allergy was higher in adults than in children^58^. The reason for this phenomenon may be due to the relative increase in cumulative prevalence with increasing age and duration of exposure.

As one of the eight major food allergens, the risk of sensitization to wheat cannot be ignored and several severe allergic reactions, even leading to shock^59^ and cardiac arrest^60^, have been reported in cases. In addition, a recent study showed that 37% of food-induced severe allergic reactions were induced by wheat^18^. However, the research related to wheat allergy in China is still in its infancy, and there are fewer reports and studies of related cases. The results of this study showed a high positive rate of allergic reactions induced by wheat in the Chinese allergic population. Hence, early screening for wheat allergy is essential in the Chinese population. It has been suggested that the timing of the introduction of foods into infants’ diets is related to their tolerance. Although the delayed introduction of strong allergens was strictly recommended for infants in Europe, the United States, and Australia, this protocol is thought to have led to an increase in allergy rates^61^. It is now recommended that allergen-containing foods could be appropriately introduced into infants’ diets at 17 to 26 weeks^61^.

Currently, the main approaches regarding the reduction of wheat allergy are mainly as follows: gluten-free diet, dietary substitution, and development of hypoallergenic wheat products. The gluten-free diet has limitations because it is extremely difficult to completely avoid wheat as wheat is common in many foods and its avoidance can have a negative impact on the growth of children. For dietary substitution, other staple foods such as rice, non-grain buckwheat, and quinoa instead of wheat may be adequate and effective substitutes for wheat allergy patients^62^. Moreover, physical, chemical, and biological methods can be used to reduce the allergenicity of wheat components. For example, phosphorylation and alkaline protease and papain hydrolysis significantly changed the conformational structure of the alcohol-soluble proteins, providing an idea for the development of the production of hypoallergenic wheat products^10^. It has also been found that sourdough fermentation is also an emerging technology for the production of hypoallergenic products^63^. In addition to avoidance of allergen intake being an effective form of management, allergen immunotherapy is also used clinically usually aimed at providing desensitization. At present, the most intensively researched areas of immunotherapy involve the oral immunotherapy (OIT), epicutaneous immunotherapy (EPIT), sublingual immunotherapy and modified subcutaneous immunotherapy (MSIT)^52^, whereas other strategies that are not allergen-specific include DNA-based vaccine^64^, omalizumab^65^, dupilumab^66^, herbal medicine^67^, probiotics and potential combination therapies^68^. Furthermore, if allergy sufferers have severe symptoms after accidental ingestion of wheat allergens, they need emergency medical attention or medication to relieve them, for example, antihistamines to reduce hives and swelling. It is recommended people with severe food allergies carry antihistamines and take them as soon as the allergy appears, and self-injected epinephrine with them to be applied in case of severe reactions^52^.

Although this meta-analysis provides the current status of wheat allergen positivity in the Chinese allergic population, it has important implications for the management of allergic patients. However, several potential limitations should be carefully considered when interpreting the study results. First, only mainstream Chinese and English databases were searched in this study, so it cannot be excluded that some publications in other non-mainstream databases were not searched. The languages of the retrieved publications were limited to English and Chinese, and this selection bias was difficult to avoid due to the language ability of the authors. After the exclusion of the literature retrieved from the English and Chinese databases, the 13 included publications were all published in Chinese databases, indicating that studies on wheat allergen positivity in Chinese allergic populations were more often published in Chinese databases. Therefore, in future studies, the inclusion of databases in other languages needs to be considered along with the expansion of searches in Chinese databases to alleviate this limitation. Second, the assessment methods of the included studies only involved sIgE and SPT, which may be the reason why the rate of positive wheat allergen testing was not affected by the assessment methods. However, it is unknown whether the positive detection rate of wheat allergens is influenced by other assessment methods, such as oral food challenge and self-reporting. Third, this incomplete survey may limit the generalizability of the findings across the country, considering that different socio-cultural and economic factors may lead to differences in prevalence between northern and southern China. Fourth, the lack of information in the included studies prevented a more detailed subgroup analysis, such as the inability to group age more carefully and to perform subgroup analysis based on allergic symptoms, which would be detrimental to understanding of the factors affecting prevalence. Finally, similar to meta-analyses of other epidemiological studies^58,69^, this study had significant heterogeneity, although random effects models were performed to obtain conservative prevalence estimates. The study population involved in this study was a clinically diagnosed allergic population, and there was no uniformity in the diagnosis of allergy, which may have led to the occurrence of higher heterogeneity due to the presence of cognitive bias. Also, the small sample size, large age group span, and large regional span in the included studies could lead to the occurrence of heterogeneity.

## Conclusions

The current study provides comprehensive and up-to-date estimates of the positive rate of wheat allergy in the Chinese allergic population from different regions of China. Overall, the positive rate of wheat allergen in the Chinese population with allergic symptoms is high. In the allergic population, the risk of sensitization varied significantly by region, with a higher risk of wheat sensitization especially in northern China. In addition, there is no publication bias in the results. To ensure the accuracy of the result, we will need to include more case studies, perform subgroup analysis concerning age, gender, and other factors, and improve this evidence base by using standardized methods to assess wheat-induced allergic disease across the population.

## Supplementary Information

Supplementary Information.

## Data Availability

Data is contained within the article. Additional data can be requested by contacting Lin Zhou (zhoulin@symc.edu.cn).

## References

[1] Cianferoni A (2016). Wheat allergy: Diagnosis and management. J. Asthma Allergy.

[2] Patel, N. & Samant, H. in *StatPearls* (StatPearls Publishing Copyright © 2022, StatPearls Publishing LLC., 2022).

[3] Fasano A, Sapone A, Zevallos V, Schuppan D (2015). Nonceliac gluten sensitivity. Gastroenterology.

[4] Cianferoni A, Spergel JM (2009). Food allergy: Review, classification and diagnosis. Allergol. Int..

[5] Ricci G (2019). Wheat allergy in children: A comprehensive update. Medicina (Kaunas).

[6] Ortiz C, Valenzuela R, Lucero AY (2017). Celiac disease, non celiac gluten sensitivity and wheat allergy: Comparison of 3 different diseases triggered by the same food. Rev. Child. Pediatr..

[7] Tranquet O, Larre C, Denery-Papini S (2020). Allergic reactions to hydrolysed wheat proteins: Clinical aspects and molecular structures of the allergens involved. Crit. Rev. Food Sci. Nutr..

[8] Sabenca C (2021). Wheat/gluten-related disorders and gluten-free diet misconceptions: A review. Foods.

[9] Taraghikhah N (2020). An updated overview of spectrum of gluten-related disorders: Clinical and diagnostic aspects. BMC Gastroenterol..

[10] Xue L (2019). Phosphorylation and enzymatic hydrolysis with alcalase and papain effectively reduce allergic reactions to gliadins in normal mice. J. Agric. Food Chem..

[11] Zuidmeer L (2008). The prevalence of plant food allergies: A systematic review. J. Allergy Clin. Immunol..

[12] Keet CA (2009). The natural history of wheat allergy. Ann. Allergy Asthma Immunol..

[13] Poole JA (2006). Timing of initial exposure to cereal grains and the risk of wheat allergy. Pediatrics.

[14] Noda R (2010). Prevalence of food allergy in nursery school (nationwide survey). Jpn. J. Food Allergy.

[15] Nwaru BI (2014). Prevalence of common food allergies in Europe: A systematic review and meta-analysis. Allergy.

[16] Weng T, Di Z (2021). Allergen analysis of atopic dermatirtis and urticaria in 992 children. Med. Recapitulate.

[17] Zheng L (2021). Analysis on quantification detection and epidemic characteristics among 20556 cases of food allergens and 1943 cases of inhalant allergens in children. Chongqing Med..

[18] Jiang N, Yin J, Wen L, Li H (2016). A retrospective study of 1952 severe allergic reactions in a Chinese population: Clinical features, triggers and treatment. Chin. J. Allergy Clin. Immunol..

[19] Page MJ (2021). The PRISMA 2020 statement: An updated guideline for reporting systematic reviews. BMJ.

[20] Ferris K (2022). How to interpret skin prick tests and serum-specific IgE in children and young people with food allergy. Arch. Dis. Child. Educ. Pract. Ed..

[21] Gao W, Li J, Jiang R, Jiang Z, Zhu M (2007). Determination of specific IgE and total IgE of176cases of children with anaphylactoid purpura. Chin. J. Lab. Diagn..

[22] Zhang C, Wei J, Yang P, Yuan X (2016). The clinical significance of food specific IgE detection in Infants with asthma. Med. Recapitulate.

[23] Li Y, Yang J, Xia Y (2021). New progress and clinical application of allergen detection. Chin. Pediatr. Integr. Tradit. Western Med..

[24] Ma Z (2021). Time trends of childhood food allergy in China: Three cross-sectional surveys in 1999, 2009, and 2019. Pediatr. Allergy Immunol..

[25] Bao C (2022). Study on distribution and characteristics of TCM syndromes in 232 adult patients with aute exacerbation of allergic asthma. J. Tradit. Chin. Med..

[26] Cao W (2016). Detection and analysis of different methods of allergens detection in systemic contact dermatitis. Chin. J. Dermatovenereol. Integr. Tradit. Western Med..

[27] Chen Z (2021). Study on the Correlation Between Food Allergen Mix Test (fx5e) and the Diagnosis and Clinical Characteristics of Food Allergy in Children.

[28] Du W, Zhang Q, Zhang W, Wang S (2022). Results of serum detection in 404 adult patients with respiratory allergic diseases. Chin. J. Pract. Med..

[29] Li J, Li Y, Wang C, Wang S (2020). Analysis on the result of allergen detection of 804 cases of children with allergic diseases. China Med. Pharm..

[30] Li W (2020). Analysis of allergen-specific IgE test results in 2 005 adults with allergic diseasesin Guangzhou. Chin. J. Immunol..

[31] Liu F, Hu C, Zhang Y (2022). Analysis of allergens of respiratory allergic diseases in children in Yulin area. Hainan Med. J..

[32] Qin P, Zhang L, Li S, Jin L, Tu C (2014). Analysis of allergens among 121 children with allergic diseases in Dalian. China J. Lepr. Skin Dis..

[33] Rina LX, Wang Y, Wang A, Fu Q (2021). Analysis of allergrn test results in 1440 children with allergic rhinitis in Ordos area of inner Mongolia. Chin. Pediatr. Integr. Tradit. Western Med..

[34] Song X (2022). Characteristics and changes of sensitization patterns of major allergens in children from2010 to 2020 in a hospital of pediatric in Beijing. Chin. J. Prev. Med..

[35] Sun Z (2008). Analysis of the Allergens with Allergic Diseases in DaLian.

[36] Xu Y, Xu R, Huang M (2013). Prevalence of allergy in children in Quanzhou city. Lab. Med. Clin..

[37] Zhao X, Zhang J, Xu N, Huang H, Yu B (2019). Clinical analysis of skin prick test results in 2560 patients with allergic dermatitis in Shenzhen. Hainan Medi. J..

[38] Jiang N, Yin J, Wen L (2014). Aspirin-Related wheat-dependent exercise induced anaphylaxis:a retrospective analysis of 20 cases. Chin. J. Allergy Clin. Immunol..

[39] Qun G (2023). Investigations on incidence and relevant factors of allergies in 5725 urban pregnant women: A cohort study in China. BMC Public Health.

[40] Silverberg JI, Hanifin J, Simpson EL (2013). Climatic factors are associated with childhood eczema prevalence in the United States. J. Invest. Dermatol..

[41] Koplin JJ (2016). Polymorphisms affecting vitamin D-binding protein modify the relationship between serum vitamin D (25[OH]D3) and food allergy. J. Allergy Clin. Immunol..

[42] Telloli C, Chicca M, Leis M, Vaccaro C (2016). Fungal spores and pollen in particulate matter collected during agricultural activities in the Po Valley (Italy). J. Environ. Sci. (China).

[43] He J (2008). Investigation on dermatitis caused by parasitic acarid in household wheat in some areas of gansu province. Endem. Dis. Bull..

[44] Chung BY, Cho YS, Kim HO, Park CW (2016). Food allergy in Korean patients with chronic urticaria. Ann. Dermatol..

[45] KA, P. *et al.* Food allergy and allergic rhinitis in 435 asian patients - A descriptive review. *The Medical journal of Malaysia* **72**, 215–220 (2017). 28889132

[46] Wang Y (2018). Asian children living in Australia have a different profile of allergy and anaphylaxis than Australian-born children: A state-wide survey. Clin. Exp. Allergy J. Br. Soc. Allergy Clin. Immunol..

[47] Jones SM, Magnolfi CF, Cooke SK, Sampson HA (1995). Immunologic cross-reactivity among cereal grains and grasses in children with food hypersensitivity. J. Allergy Clin. Immunol..

[48] Nilsson N (2018). Grass-allergic children frequently show asymptomatic low-level ige co-sensitization and cross-reactivity to wheat. Int. Arch Allergy Immunol..

[49] Sander I (1997). Differentiation between cosensitization and cross-reactivity in wheat flour and grass pollen-sensitized subjects. Int. Arch Allergy Immunol..

[50] Liu W (2023). A meta-analysis of the prevalence of wheat allergy worldwide. Nutrients.

[51] Foong RX, Dantzer JA, Wood RA, Santos AF (2021). Improving diagnostic accuracy in food allergy. J. Allergy Clin. Immunol. Pract..

[52] Sicherer SH, Sampson HA (2018). Food allergy: A review and update on epidemiology, pathogenesis, diagnosis, prevention, and management. J. Allergy Clin. Immunol..

[53] Varshney P (2011). A randomized controlled study of peanut oral immunotherapy: Clinical desensitization and modulation of the allergic response. J. Allergy Clin. Immunol..

[54] Bedoret D (2012). Changes in antigen-specific T-cell number and function during oral desensitization in cow’s milk allergy enabled with omalizumab. Mucosal Immunol..

[55] Syed A (2014). Peanut oral immunotherapy results in increased antigen-induced regulatory T-cell function and hypomethylation of forkhead box protein 3 (FOXP3). J. Allergy Clin. Immunol..

[56] Lin J (2012). A bioinformatics approach to identify patients with symptomatic peanut allergy using peptide microarray immunoassay. J. Allergy Clin. Immunol..

[57] Bunyavanich S, Schadt EE (2015). Systems biology of asthma and allergic diseases: A multiscale approach. J. Allergy Clin. Immunol..

[58] Wang J (2022). Multi-perspective observation on the prevalence of food allergy in the general Chinese population: A meta-analysis. Nutrients.

[59] Liu, X., Shang, L., Liang, X. Experience in the rescue of a case of flour allergy-induced cardiac arrest, in *The 17th World Disaster and Emergency Medicine Academic Conference and the 14th National Emergency Medicine Academic Annual Conference*, 2 (2011).

[60] Liu Y (1997). 1 case of allergic shock caused by wheat flour. J. Jinzhou Med. Univ..

[61] Czaja-Bulsa G, Bulsa M (2017). What do we know now about IgE-mediated wheat allergy in children?. Nutrients.

[62] Zhao J (2021). Extraction of total wheat (*Triticum aestivum*) protein fractions and cross-reactivity of wheat allergens with other cereals. Food Chem..

[63] Fu W, Rao H, Tian Y, Xue W (2020). Bacterial composition in sourdoughs from different regions in China and the microbial potential to reduce wheat allergens. LWT.

[64] Srivastava KD (2016). Investigation of peanut oral immunotherapy with CpG/peanut nanoparticles in a murine model of peanut allergy. J. Allergy Clin. Immunol..

[65] MacGinnitie AJ (2017). Omalizumab facilitates rapid oral desensitization for peanut allergy. J. Allergy Clin. Immunol..

[66] Bauer RN, Manohar M, Singh AM, Jay DC, Nadeau KC (2015). The future of biologics: Applications for food allergy. J. Allergy Clin. Immunol..

[67] Wang J (2015). Safety, clinical, and immunologic efficacy of a Chinese herbal medicine (Food Allergy Herbal Formula-2) for food allergy. J. Allergy Clin. Immunol..

[68] Tang ML (2015). Administration of a probiotic with peanut oral immunotherapy: A randomized trial. J. Allergy Clin. Immunol..

[69] Patsopoulos NA, Evangelou E, Ioannidis JP (2008). Sensitivity of between-study heterogeneity in meta-analysis: Proposed metrics and empirical evaluation. Int. J. Epidemiol..

