# Supplementary material for: Positive rate of wheat allergens in the Chinese allergic population: a systematic review and meta-analysis
Source: Sci Rep. 2023 Jun 29;13:10579. doi: 10.1038/s41598-023-37648-2 (PMC10310853; doi:10.1038/s41598-023-37648-2)
Supplement: Supplementary file 1 — Supplementary Information. [file 41598_2023_37648_MOESM1_ESM.doc]

Cochrane

((Allergic diseases):ab,ti,kw OR (Anaphylactic Reaction):ab,ti,kw OR (Anaphylactic Reactions):ab,ti,kw OR (Reaction, Anaphylactic):ab,ti,kw OR (Shock, Anaphylactic):ab,ti,kw OR (Anaphylactic Shock):ab,ti,kw OR (Anaphylactoid Shock):ab,ti,kw OR (Shock, Anaphylactoid):ab,ti,kw OR (Anaphylactoid Reaction):ab,ti,kw OR (Anaphylactoid Reactions):ab,ti,kw OR (Reaction, Anaphylactoid):ab,ti,kw) and ((Wheat Allergens):ab,ti,kw OR (Wheat Allergen):ab,ti,kw OR (Allergen, Wheat):ab,ti,kw OR (Wheat,Allergen):ab,ti,kw OR (Allergens):ab,ti,kw) and ((Positive rate):ab,ti,kw OR (Positive detection rate):ab,ti,kw OR (detectable rate):ab,ti,kw OR (detection rate):ab,ti,kw) AND ((China:ab,ti,kw) OR (Chinese:ab,ti,kw))

Embase

('Allergic diseases':ab,ti OR 'Anaphylactic Reaction':ab,ti OR 'Anaphylactic Reactions':ab,ti OR 'Reaction, Anaphylactic':ab,ti OR 'Shock, Anaphylactic':ab,ti OR 'Anaphylactic Shock':ab,ti OR 'Anaphylactoid Shock':ab,ti OR 'Shock, Anaphylactoid':ab,ti OR 'Anaphylactoid Reaction':ab,ti OR 'Anaphylactoid Reactions':ab,ti OR 'Reaction, Anaphylactoid':ab,ti) and ('Wheat Allergens':ab,ti OR 'Wheat Allergen':ab,ti OR 'Wheat':ab,ti OR 'Allergen':ab,ti OR 'Allergens':ab,ti) and ('Positive rate':ab,ti OR 'Positive detection rate':ab,ti OR 'detectable rate':ab,ti OR 'detection rate':ab,ti) and ('China ':ab,ti,kw OR 'Chinese ':ab,ti,kw)

WOS

(Allergic diseases OR Anaphylactic Reaction OR Anaphylactic Reactions OR Reaction, Anaphylactic OR Shock, Anaphylactic OR Anaphylactic Shock OR Anaphylactoid Shock OR Shock, Anaphylactoid OR Anaphylactoid Reaction OR Anaphylactoid Reactions OR Reaction, Anaphylactoid) and (Wheat Allergens OR Wheat Allergen OR Wheat OR Allergen OR Allergens) and (Positive rate OR Positive detection rate OR detectable rate OR detection rate ) and (China OR Chinese)

PubMed

("Allergic diseases "[Mesh]) OR ((((((((((Anaphylactic Reaction[Title/Abstract]) OR (Anaphylactic Reactions[Title/Abstract])) OR (Reaction, Anaphylactic[Title/Abstract])) OR (Shock, Anaphylactic[Title/Abstract])) OR (Anaphylactic Shock[Title/Abstract])) OR (Anaphylactoid Shock[Title/Abstract])) OR (Shock, Anaphylactoid[Title/Abstract])) OR (Anaphylactoid Reaction[Title/Abstract])) OR (Anaphylactoid Reactions[Title/Abstract])) OR (Reaction, Anaphylactoid[Title/Abstract])) and (“Wheat Allergens”[Mesh]) OR ((((Wheat Allergen [Title/Abstract]) OR (Wheat[Title/Abstract]) OR (Allergen[Title/Abstract]) OR (Allergens [Title/Abstract])) and （“Positive rate” [Mesh]） OR(((( Positive detection rate[Title/Abstract]) OR (detectable rate[Title/Abstract]) OR (detection rate[Title/Abstract])) and ( “China” [Mesh] OR (Chinese [Title/Abstract]))

Chinese Database

For China National Knowledge Infrastructure (CNKI), Chongqing VIP (CQVIP), WAN-FANG DATA and Sino Med, we conducted literature searches using the corresponding Chinese search form.
